# Effect of Localized Mechanical Indentation on Skin Water Content Evaluated Using OCT

**DOI:** 10.1155/2011/817250

**Published:** 2011-08-04

**Authors:** Abhijit A. Gurjarpadhye, William C. Vogt, Yajing Liu, Christopher G. Rylander

**Affiliations:** ^1^School of Biomedical Engineering and Sciences, Virginia Polytechnic Institute and State University, Blacksburg, VA 24061, USA; ^2^Department of Mechanical Engineering, Virginia Polytechnic Institute and State University, Blacksburg, VA 24061, USA

## Abstract

The highly disordered refractive index distribution in skin causes multiple scattering of incident light and limits optical imaging and therapeutic depth. We hypothesize that localized mechanical compression reduces scattering by expulsing unbound water from the dermal collagen matrix, increasing protein concentration and decreasing the number of index mismatch interfaces between tissue constituents. A swept-source optical coherence tomography (OCT) system was used to assess changes in thickness and group refractive index in *ex vivo* porcine skin, as well as changes in signal intensity profile when imaging *in vivo* human skin. Compression of *ex vivo* porcine skin resulted in an effective strain of −58.5%, an increase in refractive index from 1.39 to 1.50, and a decrease in water volume fraction from 0.66 to 0.20. *In vivo* OCT signal intensity increased by 1.5 dB at a depth of 1 mm, possibly due to transport of water away from the compressed regions. These finding suggest that local compression could be used to enhance light-based diagnostic and therapeutic techniques.

## 1. Introduction

A multitude of optical imaging and therapeutic techniques are being developed for the diagnosis and treatment of many human diseases, such as cancer. Soft tissues are complex heterogeneous materials, comprised of water, proteins, lipids, and cells. Skin, in particular, is composed of 70–80% water (in bound and unbound states) [1] and the remaining constituents are primarily proteinaceous structures such as collagen. The refractive index of collagen is approximately 1.55 at 1310 nm, significantly larger than that of water, 1.32 at 1310 nm [2, 3]. This heterogeneous environment contains many interfaces with mismatched refractive indices, leading to multiple scattering of incident light within the tissue, thereby decreasing light penetration depth. Shallow penetration depth is one of the main drawbacks limiting clinical effectiveness of many optical procedures. The optical penetration depth in turbid epithelial layers such as skin is generally less than 3 mm in the Vis-NIR spectrum [4, 5]. 

Tissue optical clearing is an emerging approach that explores reversible modification of scattering and absorption within naturally turbid tissues in a controlled manner. This approach permits delivery of near-collimated light deeper into tissue, potentially improving the capabilities of optical diagnostic and therapeutic techniques. Tissue optical clearing has the potential to increase the imaging depth, resolution, and contrast of optical diagnostic tools, such as optical coherence tomography (OCT) [6], optoacoustic tomography [7], fluorescence microscopy [8], and bioluminescence tomography [9]. This approach also has the potential to enhance therapeutic treatments such as laser hair removal [10] and laser lipolysis [11] by decreasing the optical scattering strength of overlying healthy tissue prior to and during laser irradiation. 

Tissue optical clearing research has focused on delivering exogenous hyperosmotic chemicals, such as glycerol and dimethyl sulfoxide, to reduce tissue light scattering and increase optical clarity. There are numerous reports of methods, applications, and potential mechanisms of tissue optical clearing using chemicals [12–17]. Three hypothesized mechanisms of light scattering reduction induced by exogenous chemicals have been proposed: dehydration or removal of endogenous water; replacement of interstitial or intracellular water with an exogenous chemical that better matches the refractive index of proteinaceous tissue structures; and structural modification or dissociation of collagen fibers [18, 19]. These three mechanisms, and possibly other unknown dynamic processes, may be acting synergistically or antagonistically with differing contributions dependent on tissue type, tissue vitality, optical clearing agent, and delivery method of said agent. 

Since dehydration is an important possible mechanism of chemical optical clearing, other nonchemical techniques for water redistribution, such as application of mechanical compression, should also optically clear tissue. We hypothesize that the mechanism of action for compression-based tissue optical clearing is the lateral removal of unbound water from regions of localized compression. This lateral water transport may cause the protein structure to become more closely packed, reducing the number of index mismatched interfaces and resultant light scattering. Mechanical optical clearing is a safer, noninvasive alternative to chemical optical clearing because no foreign material is delivered to the tissue and the stratum corneum is undisturbed, maintaining its function as a protective barrier. Such a technique may circumvent the drawbacks of chemical optical clearing and provide a fast controllable technique to enhance the light penetration in the tissue. 

Modification of tissue optical properties by mechanical forces has been investigated by several groups. Askar'yan [20] first demonstrated that local mechanical pressure results in deeper penetration of light in biotissue. Research led by Agrba et al. [21, 22] recently demonstrated use of mechanical compression for image contrast enhancement [22], or for tissue optical clearing to observe pathological changes in tissue [21]. Rylander et al. [23] described tissue optical clearing devices that utilize vacuum pressure over an array of translucent pins to fractionally compress tissues such as skin. Chan et al. and Shangguan et al. have reported increased absorption and scattering coefficients when uniform pressure is applied over large tissue area (*∼*100 mm^2^) [24, 25]. These results differ from those of Askar'yan [20], Agrba et al. [21, 22], and Rylander [23], but this is likely because Chan et al. and Shangguan et al. used uniform compression over a large area of tissue, while other works used local mechanical compression to induce smaller (*∼*1 mm^2^) compression zones, creating higher local pressure gradients within the compressed tissue.

## 2. Materials and Methods

In this study we performed experiments using a 1310 nm swept-source OCT system in order to better understand changes in water content and in light intensity profile within compressed tissue. We measured changes in thickness and refractive index of *ex vivo* porcine skin during air dehydration using the optical pathlength shift method [26]. We also calculated corresponding water volume fraction using the Lorentz-Lorenz equation [27], assuming that skin is a biphasic mixture composed of water and protein. We compared these values with independently calculated water volume fractions based on tissue sample weight loss measurements in order to verify that our biphasic mixture assumption is reasonable for this tissue. Next, we examined the effect of localized mechanical compression on *ex vivo* porcine skin thickness and refractive index. We again calculated transient water content using the Lorentz-Lorenz equation. Finally, we conducted *in vivo *human skin compression studies at several anatomical sites to determine the decrease in thickness of the stratum corneum (SC). This data was also used to evaluate changes in the OCT signal intensity profile. Local mechanical compression causes a curling of tissue structures towards the point of load application, thus making it difficult to choose specific structures for OCT signal intensity comparison. Additionally, tissue compaction in compressed regions may change layer thicknesses and reposition other tissue structures of interest. Therefore, a depth of 1 mm was chosen as a standard to compare the OCT signal intensity before and after compression.

### 2.1. Swept-Source Optical Coherence Tomography (OCT)

We constructed a swept-source OCT imaging system using a 20 kHz tunable laser (Santec HSL2000) with 1300 nm central wavelength and 110 nm FWHM bandwidth, following the design published by Chong et al. [28]. The axial resolution of the image was measured to be about 13 *μ*m. The OCT signal is an interferometric measurement sensitive to the backreflectance of photons from within the sample. OCT images represent intensity of light backreflected due to scattering as a function of optical depth (vertical axis) and lateral position (horizontal axis). The typical image depth limit of 1310 nm OCT in skin is 1-2 millimeters. Lateral dimensions of OCT images are limited by scanning optics and are typically several millimeters.

### 2.2. Skin Specimens

Two skin models were used for our studies. Porcine ventral skin was chosen for *ex vivo* experiments since optical and mechanical properties of pig skin are similar to those of human skin [29, 30]. Skin was acquired from a local market, was dehaired, and was removed from the subcutaneous fat layer. Samples were cut to approximately 30 mm × 30 mm, with thickness *∼*2 mm. 

Caucasian male volunteers (Age: 22–25, Fitzpatrick skin type: I–III) were recruited to study the effects of mechanical compression on *in vivo *human skin. SC layer thickness and changes in light intensity profile were measured during compression at four anatomical locations: fingertip, palm, dorsal hand, and ventral forearm.

### 2.3. Measurement of Ex Vivo Porcine Skin Thickness and Refractive Index

The optical path shift method developed by Sorin and Gray was used to simultaneously measure skin thickness and refractive index using OCT [26]. This procedure required placement of a fixed reflective surface (mirror) at the bottom of the *ex vivo* tissue specimen. OCT was used to measure the optical pathlength of the skin sample and the optical path shift of the reflector beneath the sample. 

**Figure 1:**
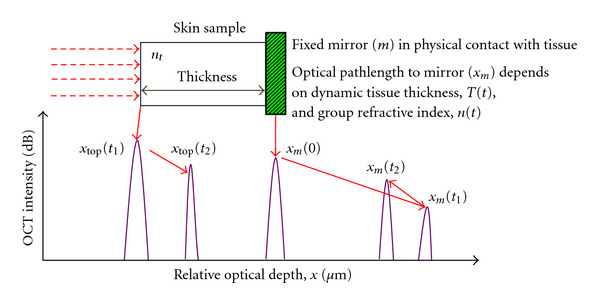
Schematic of Sorin and Gray's optical pathlength shift method. *x*_*m*_(*t*) and *x*_top_(*t*) denote optical position of the fixed mirror and top surface of the sample, respectively.

As shown in Figure 1, the relative optical position of a fixed mirror, *x*_*m*_(0) was recorded before placing the tissue sample over the mirror at time *t* > 0. When the sample was placed on the mirror, the increased optical path length created by the sample caused a shift in the optical position of the mirror, *x*_*m*_(*t*). After additionally measuring the optical position of the top surface of the sample, *x*_top_(*t*), the dynamic tissue physical thickness, *T*(*t*), and group refractive index, *n*(*t*), were calculated from the measured optical pathlengths as

(1) $$\begin{array}{cc} T\left(t\right) & =\left[x_{m}\left(t\right)-x_{\text{top}}\left(t\right)\right]-\left[x_{m}\left(t\right)-x_{m}\left(0\right)\right]\\ & =x_{m}\left(0\right)-x_{\text{top}}\left(t\right),\\ n\left(t\right) & =\frac{\left[x_{m}\left(t\right)-x_{\text{top}}\left(t\right)\right]}{T\left(t\right)}. \end{array}$$

OCT measures the group refractive index of a sample by detecting the group optical delay imposed by the sample. The actual refractive index is generally very close to that of the group index. For example, the refractive index of water at 1300 nm is different from its group refractive index by only 1.6% [31].

### 2.4. Ex Vivo Porcine Skin-Air Dehydration

*Ex vivo* porcine skin samples (*N* = 3) were placed on a fixed mirror with the hypodermis exposed to air until the sample separated from the mirror (approx. 5 hrs). A metal washer was used to hold skin samples down to prevent curling during dehydration. Room temperature was maintained between 20–25°C and relative humidity between 40–60%. OCT images were taken every 15 minutes during drying. The group refractive index and sample thickness were calculated from (1) for A-scans obtained and averaged laterally across 600 *μ*m.

### 2.5. Ex Vivo Porcine Skin-Mechanical Compression

**Figure 2:**
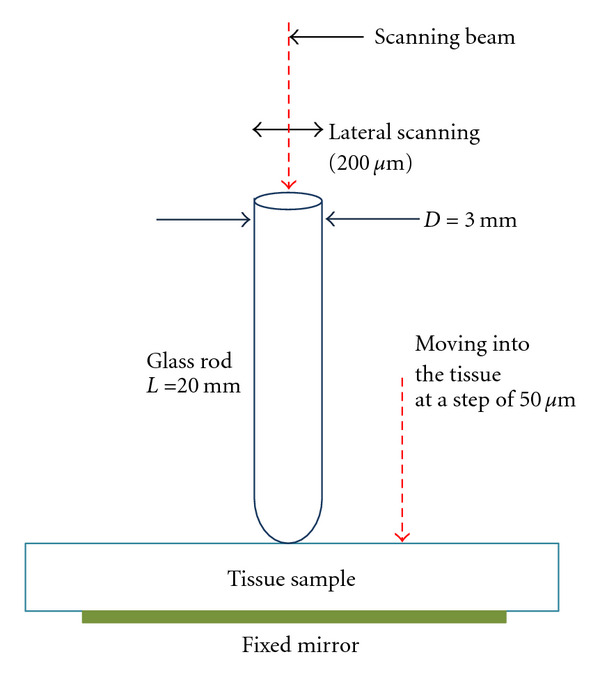
Experimental setup for tissue compression.

A hemispherically tipped glass rod (borosilicate, *n*_glass_ = 1.474) 20 mm long with a 3 mm tip diameter was used as a probe for localized skin compression as well as OCT imaging. *Ex vivo* skin specimens (*N* = 3) were placed on a fixed mirror. The rod was first positioned in light contact with the skin without significant compression. Next, the rod was translated towards the skin in increments of 50 *μ*m, applying increasing mechanical force on the tissue (Figure 2). This stepwise loading protocol allows removal of the transient viscoelastic response of the tissue, thus capturing the quasistatic mechanical deformation response. OCT A-scans averaged laterally over 200 *μ*m were obtained at each indentation step in order to measure changes in skin thickness and refractive index due to compression. The glass rod did not add any perceivable distortions to the OCT signal.

### 2.6. Ex Vivo Skin Water Content Calculation

In this study, we assume that skin is a biphasic mixture of water and proteins, primarily collagen. The refractive indices of water (*n*_water_) and protein (*n*_protein_) at 1310 nm are approximately 1.32 [2] and 1.55 [4], respectively. Let *ϕ*_water_ be the volume fraction of water in the skin and let *ϕ*_protein_ be the volume fraction of protein. From the assumption of a biphasic mixture, *ϕ*_water_ + *ϕ*_protein_ = 1, combining this constraint with the Lorentz-Lorenz rule of mixtures shown in (2), tissue water and protein concentrations were dynamically calculated during dehydration or compression [32]

(2) $$\begin{array}{cc} \frac{\left[{\left(n_{\text{skin}}\right)}^{2}-1\right]}{\left[{\left(n_{\text{skin}}\right)}^{2}+2\right]} & =\frac{\left[{\left(n_{\text{water}}\right)}^{2}-1\right]}{\left[{\left(n_{\text{water}}\right)}^{2}+2\right]}\phi_{\text{water}}\\ & \;+\frac{\left[{\left(n_{\text{protein}}\right)}^{2}-1\right]}{\left[{\left(n_{\text{protein}}\right)}^{2}+2\right]}\times \left(1-\phi_{\text{water}}\right). \end{array}$$

Tissue water content was also calculated independently by measuring the sample weight over time, *w*(*t*). Samples were weighed every 15 minutes during air dehydration. Loss of sample weight was assumed to only be due to water loss by evaporation to the environment. The skin samples were weighed again after 2 months, and assuming all the unbound water evaporated over this extended period of time, this final weight was defined as the completely dry weight, *w*(*∞*). Subtracting this dry weight from weight measurements at each time point yielded the current water weight fraction, WF(*t*), as shown in (3)

(3) $$\text{WF}\left(t\right)=\frac{w\left(t\right)-w\left(\infty \right)}{w\left(t\right)}.$$

Since the densities of water and proteins are similar, weight fraction is a reasonable approximation of volume fraction. Dynamic water weight fraction was compared with volume fraction results obtained from calculations based on the Lorentz-Lorenz equation.

### 2.7. In Vivo Human Skin Mechanical Compression and OCT Measurement of Stratum Corneum Thickness and Light Intensity Profile

Caucasian male volunteers (*N* = 3, Fitzpatrick skin type I*–*III) were recruited for *in vivo* mechanical compression studies. An OCT configuration similar to that shown in Figure 2 was used to assess changes in SC thickness and light intensity caused by compression in four anatomical regions: palm, fingertip, dorsal hand, and ventral forearm. A precompression OCT image of a small area (200 *μ*m) was obtained by placing the glass rod in light contact with the target site. Each volunteer was then asked to compress his skin against the glass rod, applying the maximum force without intolerable pain for one minute and a post-compression image was taken. Three such pre- and post-compression OCT images were taken per site per volunteer. The pain threshold stress (force per unit area) is thought to be *∼*1.1 MPa [33, 34]. An average OCT A-scan profile was generated for each anatomical site on each volunteer by averaging across the 200 *μ*m lateral scan. The optical thickness of the SC was assessed by measuring the distance between the first two consecutive intensity peaks. The SC thickness for each site of a volunteer was an average of three measurements. OCT signal intensity at a depth of *∼*1 mm (in the reticular dermis) was also evaluated using this technique.

## 3. Results

### 3.1. Ex Vivo Porcine Skin Thickness, Refractive Index, and Water Content during Air-Dehydration and Mechanical Compression

**Figure 3:**
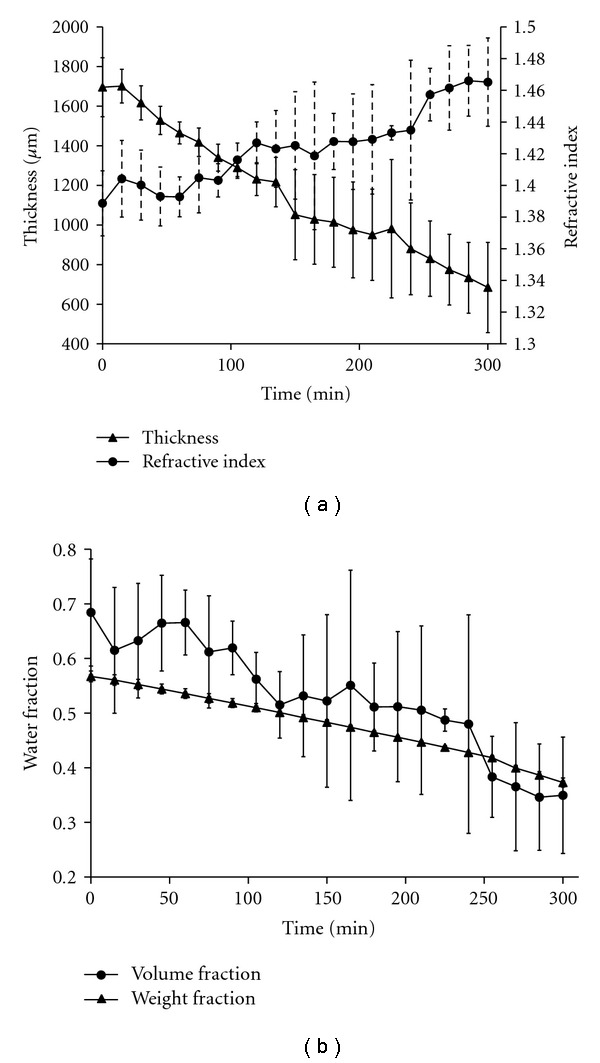
(a) *Ex vivo* porcine skin sample thickness and refractive index during air immersion. (b) Ex vivo porcine skin water volume fraction calculated from OCT data and Lorentz-Lorenz equation (circles), and skin water weight fraction calculated from weight loss measurements (triangles) during air immersion. Error bars denote 1 standard deviation.

Table 1 summarizes results from *ex vivo *air immersion and compression experiments. Dehydration of porcine skin specimens over 5 hours caused the thickness to decrease from 1700 ± 140 *μ*m to 680 ± 220 *μ*m. In order to compare water content changes between air immersed and compressed skin samples, a metric describing relative tissue thickness change is needed. Strain is a relative measure used to quantify mechanical deformation, defined as

(4) $$\text{Strain}=\frac{\Delta T}{T_{0}\;},$$

where Δ*T* is change in thickness, and *T*_0_ is original thickness. Strain is usually used in the context of deformation under a physical load, but an “effective strain” was calculated using (4) to capture the relative thickness change caused by dehydration. Dehydrated samples underwent −59.8% ± −9.6% effective strain. Tissue refractive index increased from 1.38 ± 0.02 to 1.46 ± 0.03 (Figure 3(a)). The calculated water volume fraction decreased from 0.68 ± 0.09 to 0.35 ± 0.12 over the dehydration period.  Figure 3(b) shows a positive correlation (*R*^2^ = 0.95, *P* < 0.001) between calculated water volume fraction (Lorentz-Lorenz) and independently measured water weight fractions (weight loss). This supports our assumption that skin can be represented as a biphasic mixture of water and protein, and permits use of the Lorentz-Lorenz equation for dynamic water content calculation during localized compression.

**Figure 4:**
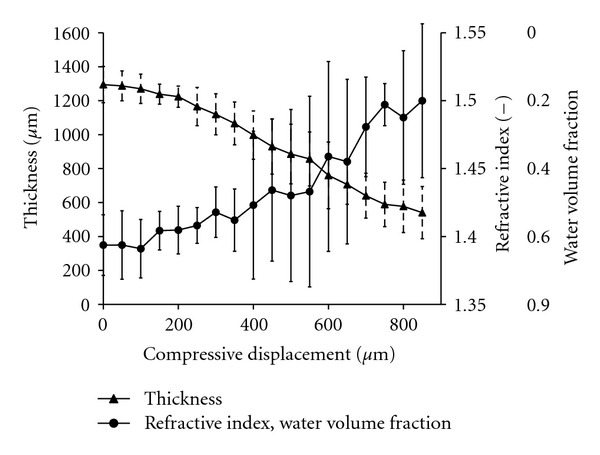
*Ex vivo* porcine skin thickness, refractive index, and water volume fraction during mechanical compression.

Mechanically compressed *ex vivo *skin specimens underwent thickness reduction from 1300 ± 100 *μ*m to 540 ± 150 *μ*m, an effective strain of −58.5% ± −8.3%.  Figure 4 shows refractive index and water content versus compressive displacement. During the initial 100 *μ*m of pin displacement, the pin had not yet made contact with skin, therefore thickness and refractive index remained unchanged. Tissue refractive index was 1.39 ± 0.02 initially and contained about 66% water. Compression to the maximum displacement increased skin refractive index to 1.50 ± 0.05 and correspondingly decreased water content to 20% water volume fraction.

### 3.2. In Vivo Human Skin Stratum Corneum Thickness and Light Intensity during Mechanical Compression

**Figure 5:**
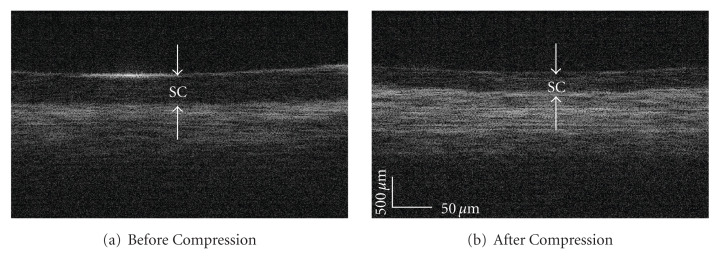
Representative OCT images and analysis of a human fingertip (a) before and (b) after compression. White arrows denote the stratum corneum.

**Figure 6:**
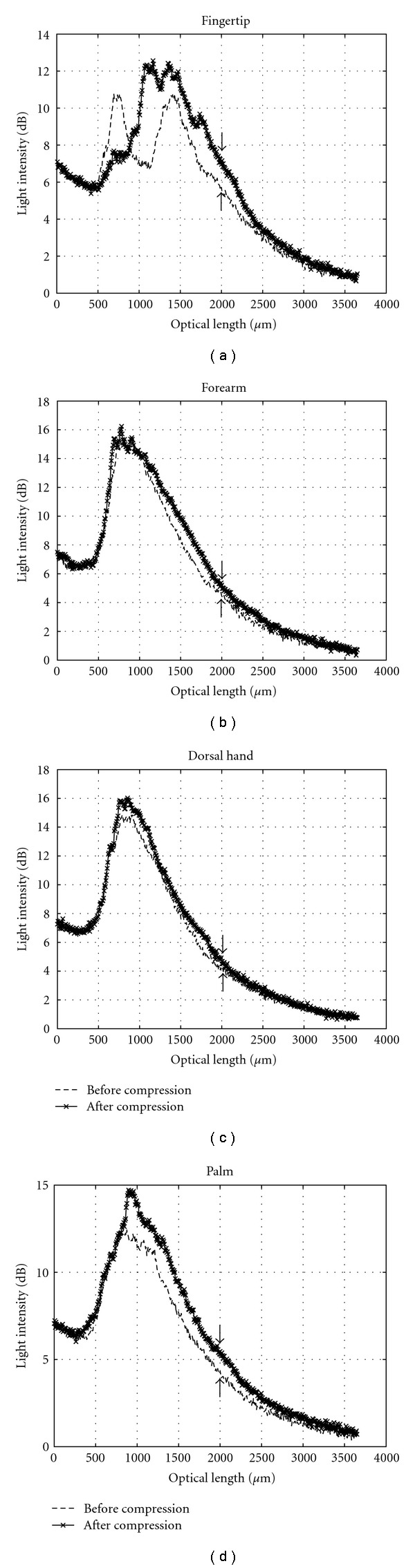
Average A-scan profiles for each of the anatomical regions studied for one representative volunteer before and after compression. Arrows denote increased signal intensity at 1 mm physical depth in skin.

*In vivo* human skin compression resulted in OCT light intensity enhancement within the dermis.  Figure 5 shows representative OCT images of one anatomical site (fingertip) before and after 1 minute of compression.  Figure 6 shows the averaged A-scan signal intensities versus optical depth for all four anatomical sites before and after compression. The first two significant peaks in the A-scan represent the skin surface and the SC-epidermal junction. The difference between these two optical depths provides the SC optical thickness. To convert this optical thickness to a physical thickness, the refractive index of the tissue was assumed to be equal to 1.5. While this value is unlikely to be valid for the bulk tissue, using a fixed value allows comparison between different samples. At sites where the skin is thick, such as the palm or fingertips, OCT images provided sufficient contrast to distinguish the SC from the epidermis. 

Table 2 summarizes results from *in vivo *human skin compression experiments. SC thickness in the fingertip (optical thickness = 220 ± 60 *μ*m) was reduced by about 50% due to compression. We observed increased OCT backreflectance in the papillary dermis. Additionally, the dermis was visible in post-compression images with the average light intensity in that region increasing by 1.5 dB at 1 mm depth. In the palm (optical thickness = 210 ± 130 *μ*m), mild enhancement of the light backscattering profile was observed in the dermis with OCT signal intensity increasing by 1.1 dB at 1 mm depth. 

In the ventral forearm, the SC thickness (optical thickness = 40 ± 10 *μ*m) reduced by about 50%, and the average OCT light intensity by about 0.8 dB. However, in the dorsal hand (optical thickness = 40 ± 10 *μ*m) compression reduced SC thickness by only 25% and increased the signal intensity by only 0.5 dB. The comparatively small reduction in SC thickness in the dorsal hand and the ventral forearm may be due to the fact that the SC is thinner in these anatomical locations. Also, each anatomical site has a different skin surface morphology, meaning the SC structure varies between these sites and may have a different mechanical response.

## 4. Discussion

The data from Table 1 illustrates that for similar effective tissue strain, air dehydration and mechanical compression produce similar changes in refractive index and water volume fraction. This suggests that mechanical compression may cause local water removal within compressed regions of skin specimens. Local water removal may increase local protein concentration. If mechanical compression expulses water from the compressed region, the intrinsic tissue optical properties of that region must change. For a biphasic mixture of water and protein, the bulk absorption coefficient of the tissue, *μ*_*a*,skin_, may be estimated from a linear rule of mixtures [35]:

(5) $$\mu_{a,\text{skin}}=\phi_{\text{water}}*\mu_{a,\text{water}}+\left(1-\phi_{\text{water}}\right)*\mu_{a,\text{protein}},$$

where *μ*_*a*,water_ is the absorption coefficient of water and *μ*_*a*,protein_ is the absorption coefficient of protein. At 1310 nm, *μ*_*a*,water_ = 1.2 cm^−1^ [36] and *μ*_*a*,protein_ = 0.25 cm^−1^ [37]. If the water volume fraction is 0.70, then *μ*_*a*,skin_ = 0.95 cm^−1^. If the volume fraction was reduced to 0.20 (as seen in our compression results) then *μ*_*a*,skin_ = 0.44 cm^−1^, a 54% reduction in absorption. This effect is particularly strong at 1310 nm because water has a high absorption peak at 1450 nm [38].

Results indicate that mechanical compression results in higher OCT signal intensity and thus better imaging capability. Mechanical compression shows strong potential to improve laser therapy and optical diagnostics in turbid tissues. Its ability to noninvasively improve OCT backreflectance intensity within the epidermis can be utilized to create devices for clinical diagnostic and therapeutic applications such as detection of epithelial cancers or laser-based thermal therapies. Skin cancers such as melanoma, basal cell carcinoma, and squamous cell carcinoma often originate from abnormal cells located at 1-2 mm beneath the skin surface. Increased optical fluence deeper in tissue due to mechanical compression may increase imaging depth in OCT, potentially enabling detection of such carcinomas. Mechanical compression could also be used for selective destruction of carcinomas or cosmetic defects in or beneath skin such as unwanted fat, hair, or wrinkles. Such compression devices could be integrated with laser delivery systems to improve laser therapies. These devices may also be incorporated into endoscopic tools to enhance internal diagnostics and laser therapeutics.

## 5. Conclusion

Localized mechanical compression of skin decreases tissue thickness and water content and increases refractive index and OCT signal intensity. Compression produces similar changes in skin thickness and optical response as compared with dehydration via air immersion. Compression likely induces water transport away from tissue regions of high compressive stress, thereby decreasing local water volume fraction and increasing the volume fraction of proteinaceous structures. This leads to increases in refractive index of the compressed region. Mechanical loading may also decrease absorption and scattering in the compressed region, particularly at 1310 nm, near the 1450 nm absorption peak of water. Decreased scattering and absorption will increase penetration depth and light intensity in the deeper regions of a target tissue. Mechanical compression has the potential to enhance light delivery for emerging optical imaging and therapeutic applications.

## Figures and Tables

**Table 1:** *Ex vivo* porcine skin results for air immersion and mechanical compression. Errors are ±1 SD.

	Air immersion		Mechanical compression	
	Before	After	Before	After
Thickness	1700 ± 140 *μ*m	680 ± 220 *μ*m	1300 ± 100 *μ*m	540 ± 150 *μ*m
Strain	—	−0.598 ± 0.096	—	−0.585 ± 0.083
Refractive index	1.36 ± 0.02	1.49 ± 0.03	1.39 ± 0.02	1.5 ± 0.05
Water volume fraction	0.68 ± 0.09	0.35 ± 0.12	0.66 ± 0.02	0.20 ± 0.05
Water weight fraction	0.57 ± 0.01	0.37 ± 0.01	—	—

**Table 2:** *In vivo* human skin measurements.

		Ventral forearm	Dorsal hand	Palm	Finger
SC Thickness (optical)	Before Compression (*μ*m)	40 ± 10	40 ± 10	210 ± 130	220 ± 160
	After Compression (*μ*m)	20 ± 10	30 ± 10	150 ± 50	110 ± 80
Increased OCT intensity at 1 mm optical depth (dB)		0.8 ± 0.2	0.5 ± 0.2	1.1 ± 0.5	1.5 ± 0.8
